# Publisher Correction: Pairwise library screen systematically interrogates *Staphylococcus aureus* Cas9 specificity in human cells

**DOI:** 10.1038/s41467-018-06029-z

**Published:** 2018-08-28

**Authors:** Josh Tycko, Luis A. Barrera, Nicholas C. Huston, Ari E. Friedland, Xuebing Wu, Jonathan S. Gootenberg, Omar O. Abudayyeh, Vic E. Myer, Christopher J. Wilson, Patrick D. Hsu

**Affiliations:** 1 Editas Medicine, 11 Hurley St., Cambridge, MA 02141 USA; 2 Whitehead Institute for Biomedical Research, Cambridge, MA 02142 USA; 3 Department of Systems Biology, Harvard, Cambridge, MA 02138 USA; 4 Department of Health Sciences and Technology, Massachusetts Institute of Technology, Cambridge, MA 02139 USA; 5 Present Address: Department of Genetics, Stanford University School of Medicine, Stanford, CA 94305 USA; 6 Present Address: Arrakis Therapeutics, 35 Gatehouse Dr., Waltham, MA 02451 USA; 7 Present Address: Department of Molecular Biophysics and Biochemistry, Yale University, New Haven, CT 06511 USA; 8 Present Address: Laboratory of Molecular and Cell Biology, Salk Institute for Biological Studies, La Jolla, CA 92037 USA

Correction to: *Nature Communications*; 10.1038/s41467-018-05391-2; published online 27 July 2018

The original HTML version of this Article incorrectly listed an affiliation of Josh Tycko as ‘Department of Genetics, Stanford University School of Medicine, Stanford, CA 94305, USA’, instead of the correct ‘Present address: Department of Genetics, Stanford University School of Medicine, Stanford, CA 94305, USA’. It also incorrectly listed an affiliation of this author as ‘Present address: Arrakis Therapeutics, 35 Gatehouse Dr., Waltham, MA, 02451, USA’.

The original HTML version incorrectly listed an affiliation of Luis A. Barrera as ‘Present address: Department of Molecular Biophysics and Biochemistry, Yale University, New Haven, CT, 06511, USA’, instead of the correct ‘Present address: Arrakis Therapeutics, 35 Gatehouse Dr., Waltham, MA 02451, USA’.

Finally, the original HTML version incorrectly omitted an affiliation of Nicholas C. Huston: ‘Present address: Department of Molecular Biophysics and Biochemistry, Yale University, New Haven, CT 06511, USA’.

This has been corrected in the HTML version of the Article. The PDF version was correct from the time of publication.

