# Supplementary material for: Pairwise library screen systematically interrogates Staphylococcus aureus Cas9 specificity in human cells
Source: Nat Commun. 2018 Jul 27;9:2962. doi: 10.1038/s41467-018-05391-2 (PMC6063963; doi:10.1038/s41467-018-05391-2)
Supplement: Supplementary file 1 — Supplementary Information [file 41467_2018_5391_MOESM1_ESM.pdf]

Supplementary Information

Pairwise library screen systematically interrogates *Staphylococcus aureus* Cas9 specificity in human cells

Josh Tycko[1,5], Luis A. Barrera[1,6], Nicholas C. Huston[1,7], Ari E. Friedland[1], Xuebing Wu[2], Jonathan S. Gootenberg[3], Omar O. Abudayyeh[4], Vic E. Myer[1], Christopher J. Wilson[1,†], Patrick D. Hsu[1,8,†]

Affiliations:

1. Editas Medicine, 11 Hurley St., Cambridge, MA 02141, USA
2. Whitehead Institute for Biomedical Research, Cambridge, MA 02142, USA
3. Department of Systems Biology, Harvard, Cambridge, MA 02138, USA
4. Department of Health Sciences and Technology, Massachusetts Institute of Technology, Cambridge, MA 02139, USA
5. Present address: Department of Genetics, Stanford University School of Medicine, Stanford, CA 94305, USA
6. Present address: Arrakis Therapeutics, 35 Gatehouse Dr., Waltham, MA 02451, USA
7. Present address: Department of Molecular Biophysics and Biochemistry, Yale University, New Haven, CT 06511, USA
8. Present address: Laboratory of Molecular and Cell Biology, Salk Institute for Biological Studies, La Jolla, CA 92037, USA

†Correspondence: patrick@salk.edu or christopher.wilson@editasmed.com

Supplementary Figures

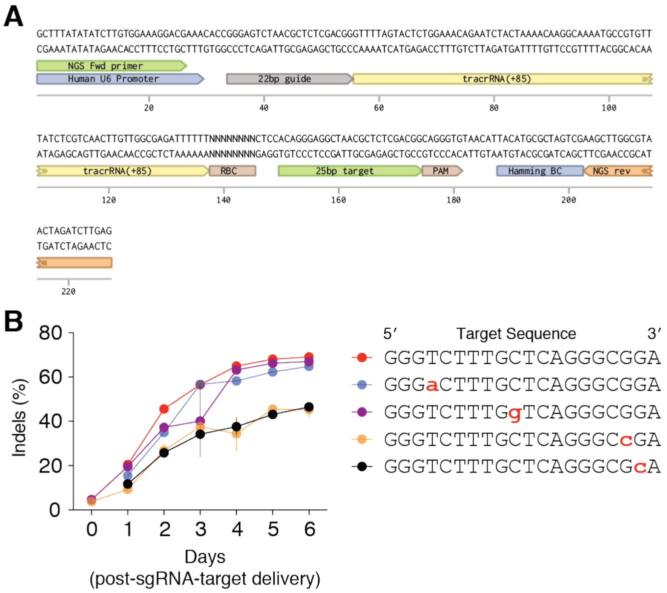

Supplementary Figure 1. SaCas9 genome editing saturates 5 days after lentiviral delivery of the pairwise guide-target cassette.

A. Sequence of the pairwise guide-target cassette with an example 22-nt spacer and target, randomized barcode (rBC), Hamming barcode (BC), and next-generation sequencing (NGS) primer-binding sites shown.
B. Temporal dynamics of SaCas9-mediated indels on 5 pairwise guide-target cassettes. SaCas9 and the pairwise cassettes were delivered by lentivirus to HEK 293T cells in individual wells (mean ± S.E.M., n = 2). Mismatched target positions are shown in red.

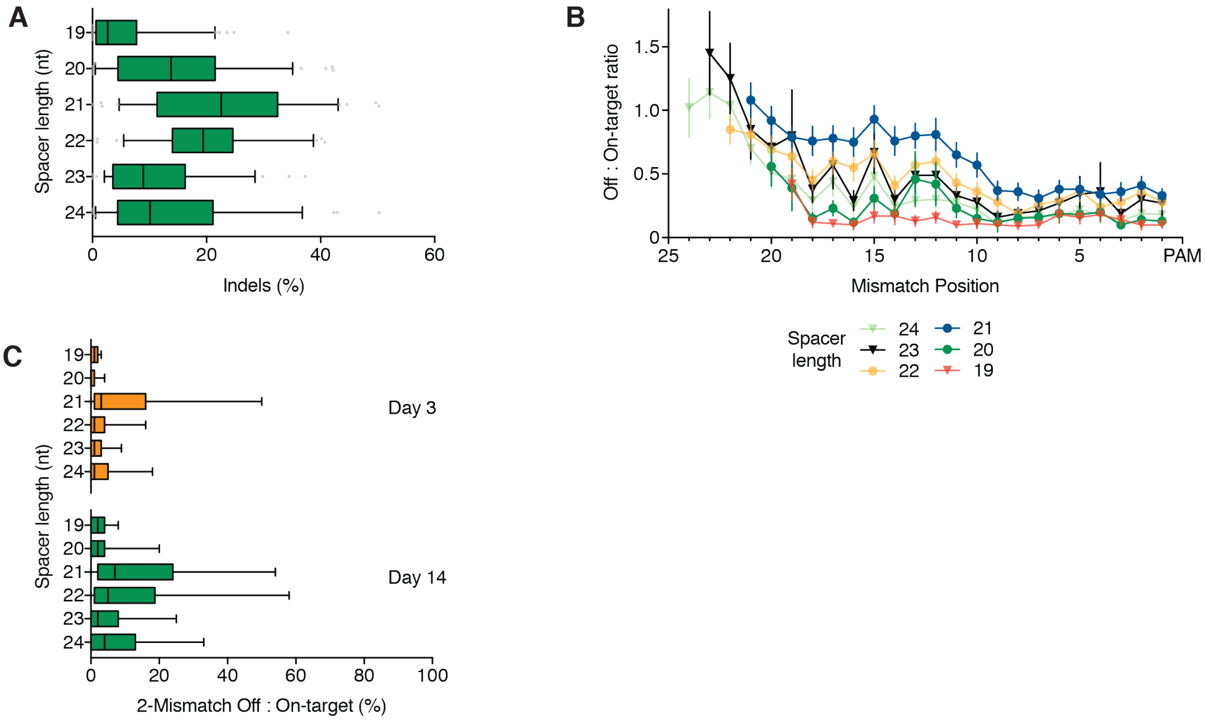

Supplementary Figure 2. Effect of spacer length on activity 14 days after SaCas9 delivery.

A. On-target indel efficiency on Day 14 for SaCas9 guide-target pairs, binned by spacer length. (n = 255 sgRNA-target pairs)

B. Average effect of sgRNA spacer length and mismatch position on SaCas9 single mismatch tolerance at Day 14. Mean ± 95% confidence interval is shown. (n = 16,545 sgRNA-target pairs)

C. Double mismatch tolerance binned by spacer length. (n = 28,708 and 23,673 sgRNA-target pairs at Day 3 and 14, respectively)

Boxes in A and C denote median and IQR, and whiskers extend to the 10th and 90th percentile.

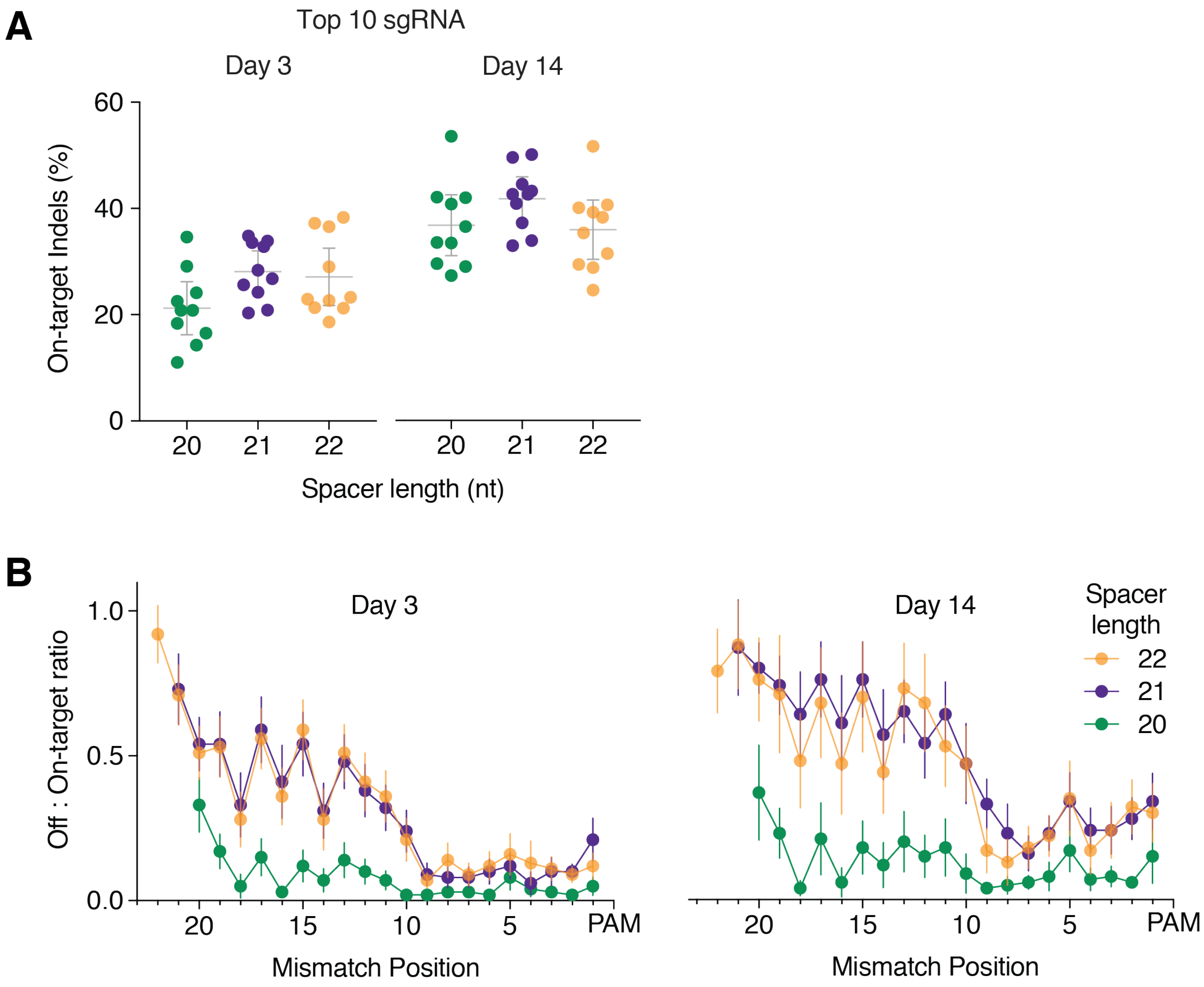

Supplementary Figure 3. Highly active sgRNAs with 20 nt spacers retain improved mismatch sensitivity.

A. Efficiency of the top 10 most active sgRNA from each spacer length. Mean ± 95% confidence interval is shown, with a dot for each sgRNA. (n = 30 sgRNA-target pairs on both days)

B. Average effect of sgRNA spacer length and mismatch position on SaCas9 single mismatch tolerance for the top 10 most active sgRNA. Mean ± 95% confidence interval is shown. (n = 2,680 and 1,808 sgRNA-target pairs at Day 3 and 14, respectively)

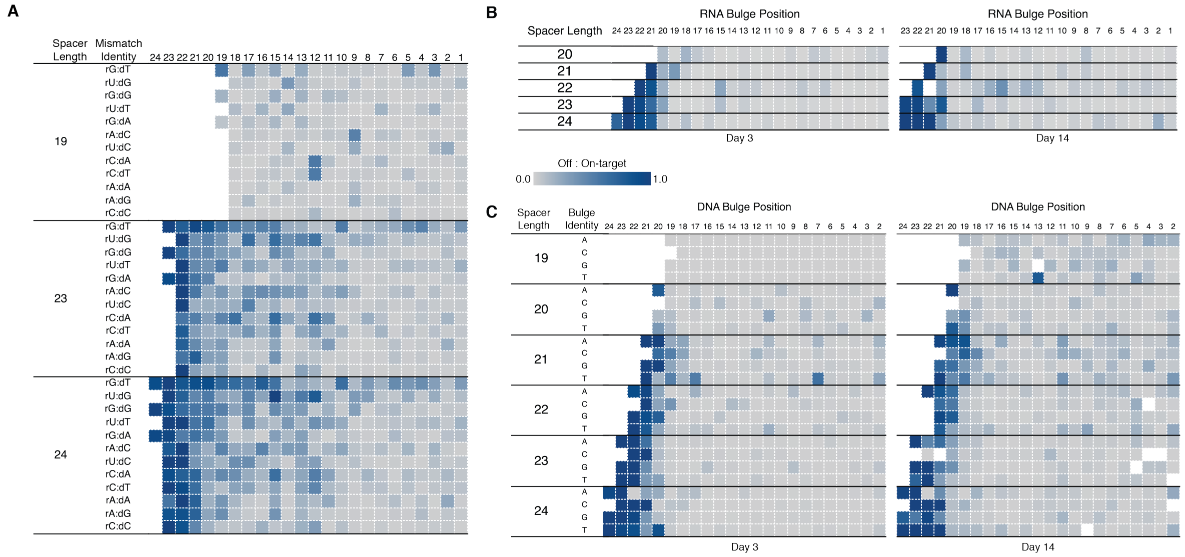

Supplementary Figure 4. SaCas9 single mismatch tolerance of 19, 23, and 24 nt sgRNA spacer lengths and low bulge tolerance.

A. Heatmap of SaCas9 single mismatch tolerance at Day 3 for each possible RNA:DNA base pair. (n = 6,398 sgRNA-target pairs)
B. SaCas9 RNA bulge tolerance was measured with all possible bulges. ‘1’ represents an extra RNA base added to the spacer at the most PAM-proximal position. (n = 327 and 339 sgRNA-target pairs on Day 3 and 14, respectively)
C. SaCas9 DNA bulge tolerance was measured with all possible bulges. ‘2’ represents an extra DNA base added to the target between the first and second-most PAM-proximal positions. (n = 1,206 and 1,316 sgRNA-target pairs on Day 3 and 14, respectively)

A

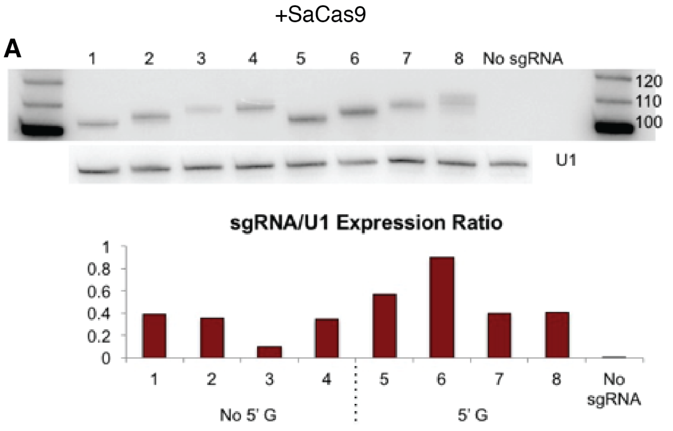

B

sgRNA	Spacer
1	ACCATAGTGAGCCCAGAA
2	CTACCATAGTGAGCCCAGAA
3	TCCTACCATAGTGAGCCCAGAA
4	CATCCTACCATAGTGAGCCCAGAA
5	GCCATAGTGAGCCCAGAA
6	GTGCCATAGTGAGCCCAGAA
7	GCGTGCCATAGTGAGCCCAGAA
8	GAGCGTGCCATAGTGAGCCCAGAA

C

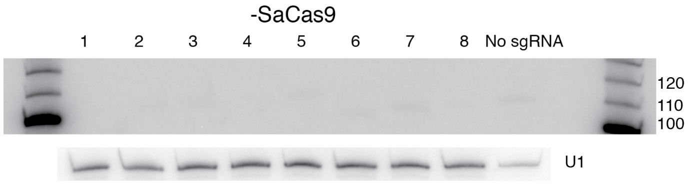

Supplementary Figure 5. Northern blot verification of sgRNA length and dependency on SaCas9 co-expression.

A. sgRNA with 18, 20, 22, or 24 nt spacers were co-transfected with SaCas9 in HEK293T cells. Small RNA was extracted and the sgRNA were detected by Northern blot, with a probe designed against the sgRNA scaffold. U1 is used as a loading control. The sgRNA sequences are the same except for the length, and the presence or absence of a G in the 5′ position.
B. sgRNA spacer sequences ranging from 18- to 24 nt without or with a 5′ 'G'.
C. The same sgRNA are hardly detectable when transfected without SaCas9.

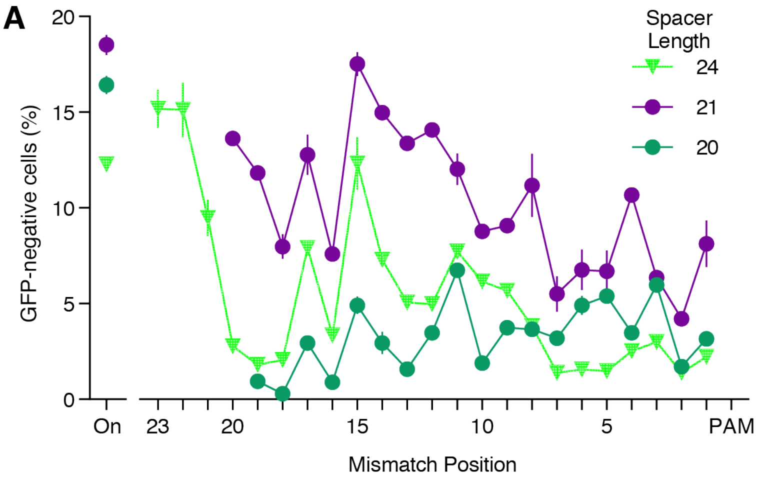

Supplementary Figure 6. Orthogonal assay validates that 20 nt spacer sgRNA are less tolerant of single mismatches.

A. GFP-targeting sgRNAs were generated such that there was an sgRNA with a single mismatch at each position. 'On' labels the on-target matched sgRNA. sgRNA expression cassettes were individually transfected into an HEK293-GFP cell line with stably integrated GFP. GFP knockout was measured by flow cytometry to quantify GFP negative cells (mean ± S.D., n = 65 sgRNAs with 2 biological replicates each).

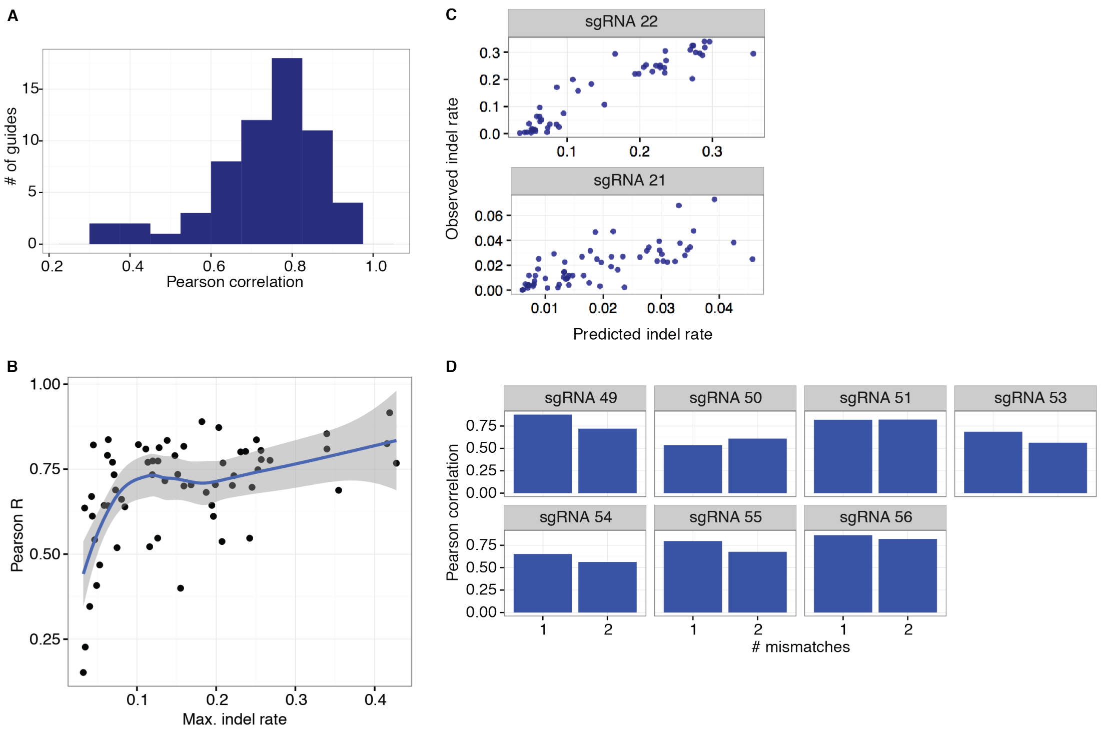

Supplementary Figure 7. Specificity score performance on screen data.

A. The score was fit to the observed off-target data for each guide in the screen, individually. The histogram shows the Pearson correlation of the score against the observed data from all library members in that guide group.
B. The score's fit was compared with the maximum indel rate observed within each guide group.
C. The score's performance is shown for two guides, one with high maximal activity (sgRNA 22) and one with very low maximal activity (sgRNA 21). Each dot represents a guide-target pair within that guide group.
D. The score's performance predicting activity at both the single and double-mismatch off-targets. sgRNA groups 49 – 56 are groups for which all single and double mismatches were included in the pairwise library screen.

Supplementary Tables

Category	Guide Groups	Spacer Lengths	Guides	Targets	Library Members
**On-target**	73	19 - 24 nt	438	545	653
**Single mismatch**	73	19 - 24 nt	438	29,417	33,417
**Double mismatch**	10	19 - 24 nt	60	43,815	50,736
**DNA bulge**	5	19 - 24 nt	30	1,591	2,377
**RNA bulge**	5	19 - 24 nt	30	452	633
**Negative control**	73	19 - 24 nt	438	530	876
**Total**					**88692**

Supplementary Table 1. Pairwise library design. Size of the categories of target sites and spacers included in the pairwise library.
