# Supplementary material for: Pairwise library screen systematically interrogates Staphylococcus aureus Cas9 specificity in human cells
Source: Nat Commun. 2018 Jul 27;9:2962. doi: 10.1038/s41467-018-05391-2 (PMC6063963; doi:10.1038/s41467-018-05391-2)
Supplement: Supplementary file 3 — Description of Additional Supplementary Files [file 41467_2018_5391_MOESM3_ESM.pdf]

## Description of Additional Supplementary Files

File Name: Supplementary Data 1
Description: **Oligonucleotides used in this study.** List of oligonucleotide sequences used as PCR primers in this study

File Name: Supplementary Data 2
Description: **Plasmids used in this study.** List of plasmid sequences used to express SaCas9 and sgRNAs, including the lentiviral pairwise library vector.

File Name: Supplementary Data 3
Description: **Pairwise library design and sequences.** 624 Table including the specific sequences of guides and targets used in the SaCas9 pairwise library screen.
